# District-level heterogeneity in overweight or obesity among women of reproductive age: A geo-spatial analysis in India

**DOI:** 10.1371/journal.pone.0290020

**Published:** 2023-08-17

**Authors:** Sampurna Kundu, Pratima Sharma, Shivani Singh, Pradeep Kumar

**Affiliations:** 1 Centre of Social Medicine and Community Health, Jawaharlal Nehru University, Delhi, India; 2 School of Health System Studies, Tata Institute of Social Sciences, Mumbai, India; 3 Specialist- Monitoring and Evaluation, India Health Action Trust (IHAT), Lucknow, India; Flinders University, AUSTRALIA

## Abstract

**Background:**

Globally by 2030, 38% of the world’s population would be overweight, and another 20% would be obese. This has led to rising concerns regarding how swiftly and substantially the world is moving towards this epidemic of "globesity". India too is facing an increased burden of overweight and obese population. The changing dietary patterns are significantly associated with the increasing prevalence of overweight/obesity and related complications, especially among women. Hence, the present study aims to observe the spatial patterns of overweight or obesity among women in reproductive age group in India and factors associated with it.

**Methods:**

The study analyzed data from a cross-sectional nationwide household survey, i.e. National Family Health Survey (NFHS-4), 2015–16. The primary outcome variable of this study was overweight/obesity among reproductive-age women, which was measured through the body mass index (BMI) of the women. Bivariate and multivariate logistic regression analysis was used to analyze the data. Additionally, for spatial analysis in terms of overweight/obesity among women in India, univariate and bivariate Moran’s I index measurements were used along with the usage of spatial regression models.

**Results:**

The value of spatial-autocorrelation for overweight or obese was 0.64, which depicts the moderately high prevalence of the overweight/obesity coverage over districts of India. The overall prevalence overweight/obesity among women in India is around 25% and higher proportion of women from urban areas (37.8%), and non-poor (33.4%) economic group reported to be overweight or obese. From spatial lag model, the lag coefficient was found to be 0.28, implying that a change in the prevalence of overweight/obesity among women in a certain district may statistically lag the prevalence of overweight/obesity by 28% in the neighbouring districts. There were significantly high clustering of overweight/obese women and non-poor wealth quintiles in 132 districts, mainly from states of Punjab, Haryana, Gujarat, Maharashtra, Kerala, Tamil Nadu, Karnataka and Andhra Pradesh. Additionally, there was high-high clustering of overweight/obese women and those who ever had caesarean in 82 districts, mostly from Kerala, Tamil Nadu, Andhra Pradesh and Karnataka.

**Conclusion:**

The spatial patterns on the prevalence of overweight and obesity in India show that the women belonging to the southern states’ districts are more overweight or obese in comparison to other states. The determinants like older age, higher education, urban residence, higher economic status are the key factors contributing to the prevalence of overweight or obesity among women in the reproductive age group. The study concludes and recommends an urgent need of interventions catering to urban women belonging to higher socio-economic status, to reduce the risks of health consequences due to overweight and obesity.

## Introduction

Health remains the key indicator of a country’s economic growth and overall development. A profound shift has been observed among major causes of disease and deaths worldwide in the last three decades [1]. Non-communicable diseases (NCDs) contribute significantly to the global disease and deaths burden, with their share gradually increasing in overall global deaths from 60.8% in 2000 to 73.6% in 2019 [2]. This has further burdened the already strained health systems. Overweight and obesity are the two main risk factors associated with NCDs such as diabetes, hypertension, strokes, and even certain cancers [3, 4].

Overweight and obesity can be defined as ’excessive fat accumulation that may impair health.’ Though it is multifactorial and largely preventable, it affects more than a third of the world’s population [5, 6]. Between 1975 to 2016, the prevalence of obesity increased three times [7]. In 2016, more than 1.9 billion adults (nearly 39%) were overweight, and more than 650 million (13%) were obese [2, 8]. The global age-standardized prevalence of obesity increased from 6.4% in 1980 to 12.0% in 2008, whereas for overweight, it increased from 24.6% to 34.4% during the same 28-year period [7]. It shows how swiftly and substantially the world is moving towards this epidemic of "globesity" [2, 7, 8]. It was estimated that by 2030, 38% of the world’s population would be overweight, and another 20% would be obese [8].

Earlier, overweight and obesity posed a major threat to high-income countries only, but lately it is becoming more prevalent and a matter of public health concern among middle, lower middle-income countries (LMIC) and low-income countries [4, 9–12]. This can be because many developing countries are undergoing epidemiological, nutritional, and economic transitions [13, 14]. India, too is undergoing a nutritional transition with an increased burden from overweight and obesity [15]. Nearly 135 million individuals in India are affected by obesity [16]. Recent data from India shows that almost 13–50% of the urban population and 8–38% of the rural population suffer from obesity [17]. Overweight and obesity are commonly assessed by an individual’s body mass index (BMI), which is defined as the weight in kilograms divided by the square of the height in meters (kg/m2). A value above 25 kg/m^2^ is described as overweight and over 30 kg/m^2^ as obese [2].

Although men may have higher rates of overweight, women have higher rates of obesity [2]. The changing dietary patterns are significantly associated with the increasing prevalence of overweight/obesity and related complications, especially among women [15–19]. Women are more prone to obesity or overweight, specifically those who belong to urban areas and are from good socio-economic status [15, 16, 20]. Obesity among women affects not only them but their future generations too as it tends to cause childhood obesity [21]. The physiological and psychological changes with childbirth also affect women’s food intake habits and nutritional status [22]. Various studies that are associated with obesity and overweight among women document age, sex, educational level, place of residence, income, availability of resources, pregnancy, parity, menopause, maternal smoking, food habits, sedentary lifestyle, watching television, etc. as some of the key factors associated with its higher prevalence [20, 23–30].

Obesity, a visible yet neglected public health concern, affects almost all age groups and often leads to the conditions that reduce overall quality of life. It is closely related to our nutritional status and day-to-day maintenance of health. However, in the Indian context, there are tiny shreds of evidence in the literature identifying the associations between these socioeconomic factors and the prevalence of overweight and obesity. Hence, this study focuses on various factors associated with India’s increasing overweight and obese reproductive age female population. We used nationally representative data to analyze the prevalence of overweight and obesity and identify various factors associated with it among women of reproductive age in India. The present study tried to answer the following questions: i) What are the determinants of overweight or obesity among reproductive-age women in India? ii) Is there any geographical clustering of overweight or obese women of reproductive-age across India? The analysis will help understand the pattern of overweight and obesity among reproductive age women across districts of India that may further help identify target areas, to engage relevant stakeholders, and develop public health interventions for reducing the burden of obesity and associated adverse consequences.

## Data and methods

The study analyzed data from a cross-sectional nationwide household survey, i.e. National Family Health Survey (NFHS-4), which provided information on health-related matters, including fertility, morbidity and mortality, family planning, and nutrition, conducted in 2015–16. The NFHS was carried out in 29 Indian states and seven union territories and yielded estimates for 640 districts for the first time. The survey adopted a two-stage stratified sampling design for the selection of the representative sample. Villages were chosen in the first step in rural areas using a Probability Proportional to Size (PPS) scheme. The second stage involved systematic sampling to choose 22 HHs. In urban areas, census enumeration blocks (CEBs) were chosen using the PPS scheme in the first stage, and 22 HHs were chosen using systematic sampling in the second stage. The detailed methodology and complete information on the survey design and data collection are published elsewhere [31]. NFHS-4 collected information from 601,509 households (HH), 699,686 women aged 15–49 years, and 112,122 men aged 15–54 years for the response rate of 98%, 97%, and 92% respectively. This study considers only those women who have complete data on the anthropometric outcomes. We have excluded women who were currently pregnant during the survey and women with a birth in two months prior to the survey as their weight would not be representative. After excluding the sample of currently pregnant and women with a birth in the last 2 months, the final sample size for this study is 454,517 women aged 15–49 years.

### Outcome variable

The primary outcome variable for this study was overweight/obesity among reproductive-age women, which was measured through the body mass index (BMI) of the women. BMI was calculated as weight in kilograms divided by height in meters squared (kg/m^2^). The study used World Health Organization classifications, BMI cut-offs were divided into those overweight/obese (BMI≥25) and those not overweight/obese (BMI<25) [32, 33].

### Exposure variables

The predictors for overweight/obesity among women were women’s age, education, caste, religion, place of residence, wealth, ever had caesarean, ever had terminated pregnancy, watching TV, and eating fast food. Women’s age was grouped into two categories: below 30 years and 30 and above years. The educational level of women was categorized as no/primary and secondary and higher education. Caste was grouped into two categories: scheduled caste/scheduled tribe (SC/ST) and Non-SC/ST. Religion was categorized as Hindu and non-Hindu (including Christian, Sikh, Buddhist/Neo-Buddhist, Jain, Jewish, Parsi/Zoroastrian, no religion, and other). Place of residence was given as rural and urban in the survey. In the survey, a household wealth index was calculated by combining household amenities, assets, and durables and categorizing households in a range from poorest to richest, corresponding to wealth quintiles from lowest to highest. Furthermore, this study divided household wealth into two categories: poor (which comprised the poorest and poorest) and non-poor (included middle, richer, and richest). Ever had caesarean was coded as ‘yes’ if women delivered by caesarean section and ‘no’ otherwise. If women ever got their pregnancy terminated, it was coded as ‘yes’ and ‘no’ if otherwise. The question regarding television asked to the women was “Do you watch television almost every day, at least once a week, less than once a week, or not at all?” The responses were coded ‘yes’ if the frequency of watching TV was every day and ‘no’ elsewise. Similarly, eating fast food was coded as yes if women ate fried food or aerated drinks daily and no, otherwise.

### Statistical analysis

Bivariate and multivariate logistic regression analysis was used to analyze the data. Additionally, for spatial analysis in terms of overweight/obesity among women in India univariate and bivariate Moran’s I index measurements were used along with the usage of spatial regression models [34, 35]. Spatial auto-correlation is being measured by using Moran’s I statistics. Spatial autocorrelation represents the extent to which data points are similar or dissimilar to their spatial neighbours [35–37]. The current analysis was controlled for complex survey design (clustering, weighting, and stratification) by using svyset command in STATA.

Univariate Moran’s I measure the spatial auto-correlation of neighborhood values around a specific spatial location. It determines the extent of spatial non-stationery and clustering present in the data. Bivariate Moran’s I examines the local correlation between a outcome variable and certain characteristics of region. While both univariate and bivariate Moran’s I aim to measure similarities and dissimilarities of spatial data, they are found to be less useful in case of uneven spatial clustering [34, 37]. The formula to calculate the Moran’s *I* statistic is as follows:

$$Univariate\;Moran’s\;I=\frac{n}{S_{O}}\times \frac{\Sigma_{i}\Sigma_{j}W_{ij}(x_{i}-\bar{X})(x_{j}-\bar{X})}{{\Sigma_{i}(x_{i}-\bar{X})}^{2}}$$

Where x is the variable of interest and $\bar{X}$ is the mean of x; n is the number of spatial units; *W*_*ij*_ is the standardized weight matrix between observation i and j with zeroes on the diagonal; and *S*_*O*_ is the aggregate of all spatial weights, i.e. *S*_*O*_ = Σ_*i*_Σ_*j*_*W*_*ij*_

$$Bivariate\;Moran’s\;I=\frac{n}{S_{O}}\times \frac{\Sigma_{i}\Sigma_{j}W_{ij}(x_{i}-\bar{X})(Y_{j}-\bar{Y})}{{\Sigma_{i}(y_{i}-\bar{Y})}^{2}}$$

Where x and y are the variables of interest; $\bar{X}$ is the mean of x; $\bar{Y}$ is the mean of y; n is number of spatial units; *W*_*ij*_ is the standardized weight matrix between observation i and j with zeroes on the diagonal; and *S*_*O*_ is the aggregate of all spatial weights, i.e. *S*_*O*_ = Σ_*i*_Σ_*j*_*W*_*ij*_.

Value of Moran’s- I ranges from −1 (indicating perfect dispersion) to +1 (perfect correlation). A zero value indicates a random spatial pattern. Negative (positive) values indicate a negative spatial autocorrelation. Positive autocorrelation indicates that points with similar attribute values are closely distributed in space, whereas negative spatial autocorrelation indicates that closely associated points are more dissimilar [38, 39].

Univariate LISA calculates the spatial-correlation of neighborhood values around the specific spatial location [36]. It determines the extent of spatial randomness and clustering present in the data. The measure (*I*_*i*_) is given by the following:

$$Univariate\;LISA:\;I_{i}=\frac{n.(x_{i}-\bar{X})}{\Sigma_{i}{\left(x_{i}-\bar{X}\right)}^{2}}\Sigma_{j}w_{ij}(x_{j}-\bar{X})$$

Bivariate LISA measures were estimated to analyze the association of certain characteristics of regions with different outcomes of child immunization [36]. The bivariate LISA is presented as below:

$$Bivariate\;LISA:\;I_{i}=\frac{n.(x_{i}-\bar{X})}{\Sigma_{i}{\left(y_{i}-\bar{Y}\right)}^{2}}\Sigma_{j}w_{ij}(y_{i}-\bar{Y})$$

Four types of spatial auto-correlation were generated based on the four quadrants of Moran’s I scatter plots which are defined as follows:

- **Hot Spots**: districts with high values, with similar neighbours (High-High).
- **Cold Spots**: districts with low values, with similar neighbours (Low-Low).
- **Spatial Outliers**: districts with high values, but with low-value neighbours (High-Low) and districts with low values, but with higher values of neighbours (Low-High).

The spatial weights W_ij_ are non-zero when i and j are neighbors, else it remains zero [37]. The weight used in the analysis is Queen Contiguity weights which represents whether spatial units share the boundary or not. If the set of boundary points of unit I is denoted by band (i), then the Queen Contiguity Weight is defined by:

$$W_{ij}=\left\{ \begin{array}{l} 1,\;bnd(i)\cap bnd(j)\neq \emptyset \\ 0,\;bnd(i)\cap bnd(j)\neq \emptyset \end{array}\right.$$

However, this allows the possibility that spatial units share only a single boundary point (such as a shared corner point on a grid of spatial units). Hence a stronger condition is to require that some *positive* portion of their boundary be shared.

In order to determine the significant correlates of overweight/obesity among women in India, a set of regression models had been used. Spatial ordinary least square (OLS) regression model was used to see the extent of autocorrelation in the error term. Since the OLS confirmed spatial autocorrelation in its error term for the dependent variable, we further estimated spatial lag model (SLM) and spatial error model (SEM) [38, 39]. The underlying assumption of a spatial lag model is that the observations of the outcome variable are affected in the neighborhood areas whereas the spatial error model is used to consider the effect of those variables which are absent in the regression model but had an effect on the outcome variable. The basic difference between the two models is that the spatial lag model unlike spatial error model does not consider the spatial dependence of the error term.

The basic equation for OLS is as follows:

Y=α+βX+Ɛ

Where Y is the outcome variable, X is the vector of predictor variables and α is the model intercept and β is the corresponding coefficient vector.

Spatial lag model suggests that the units are spatially dependent to each other and lagging to each in the nearby spatial locations [38, 39]. A typical spatial lag model can be written as follows:

$$Y_{i}=\delta \sum_{j\neq 1}W_{ij}Y_{j}+\beta X_{j}+\varepsilon_{j}$$

Here *Y*_*i*_ denotes overweight/obesity among women for the *i*^*th*^ district, δ is the spatial autoregressive coefficient, *W*_*ij*_ denotes the spatial weight of proximity between district i and j, *Y*_*j*_ is overweight/obesity among women in the *j*^*th*^ district, *β*_*j*_ denotes the coefficient, *X*_*j*_ is the predictor variable and ε_j_ is the residual.

Spatial error model on the other hand, considers the contribution of omitted variables which are not included in the model but can have significant effect in the analysis [38, 39]. A Spatial Error Model (SEM) is expressed as follows:

$$Y_{i}=\beta X_{j}+\lambda \sum_{j\neq 1}W_{ij}Y_{j}\varepsilon_{j}+\varepsilon_{i}$$

Here, *Y*_*i*_ denotes overweight/obesity among women for the *i*^*th*^ district, λ is the spatial autoregressive coefficient, *W*_*ij*_ denotes the spatial weight of proximity between district i and j, *Y*_*j*_ is overweight/obesity among women in the *j*^*th*^ district, *β*_*j*_ denotes the coefficient, *X*_*j*_ is the predictor variable and *ε*_*i*_ is the residual.

## Results

**Table 1** depicts the socio-demographic profile of the study population. The evidences show that one in every four women (24.9%) of age group 15–49 years was either overweight or obese. Majority of the women belonged to age group 30–49 years (64.6%), 48.1% had either no education or were educated up to primary, 70.8% belonged to non-SC/ST category and 81.7% belonged to Hindu group. Almost two-third (66.2%) women lived in rural areas, and more than one-third (37.3%) belong to poor economic group. Only 6.8% women ever had a caesarean and 15.9% had a terminated pregnancy. More than three-fourth women (76.2%) watched TV and more than half (52.5%) consume fast-food.

**Table 1:** Socio-demographic profile of the study population in India, 2015–16.

Variables	N = 454517
	[n(weighted %)]
**Overweight/obesity**	
No	351294 (75.1)
Yes	103223 (24.9)
**Age (in years)**	
15–29	157896 (35.4)
30–49	296621 (64.6)
**Education**	
No or primary	227660 (48.1)
Secondary or higher	226857 (51.9)
**Caste**	
SC/ST	159292 (29.2)
Non-SC/ST	295225 (70.8)
**Religion**	
Hindu	346657 (81.7)
Non-Hindu	107860 (18.3)
**Place of residence**	
Urban	128248 (33.8)
Rural	326269 (66.2)
**Wealth**	
Poor	185605 (37.3)
Non-Poor	268912 (62.7)
**Ever had caesarean**	
No	427861 (93.2)
Yes	26656 (6.8)
**Ever had terminated pregnancy**	
No	381690 (84.1)
Yes	72827 (15.9)
**Watching TV**	
No	117848 (23.8)
Yes	336669 (76.2)
**Eats fast food**	
No	219502 (47.5)
Yes	235015 (52.5)

**Table 2** depicts the socio-demographic characters associated with overweight/obese women. The bivariate analysis shows that a higher proportion of women from older age-group (30.5%), or with secondary or higher education (30.1%), or from SC/ST group (27.7%) were overweight/obese. Further, more women from non-Hindu group (30.3%) as compared to Hindu group (23.7%) were overweight or obese. A higher proportion of women from urban areas (37.8%), and non-poor (33.4%) economic group reported to be overweight or obese. About 31.1% women who ever had caesarean and 28.9% who ever had a pregnancy terminated were overweight/obese. The results from logistic regression analysis depicts that women from older age-groups (30–49 years) were almost three times (AOR = 2.9, *p<0*.*01*) more likely to be overweight/obese as against their younger counterparts. Women with secondary or higher education had higher odds to be overweight/obese as compared to women with no or up to primary education (AOR = 1.29, *p<0*.*01*). Women from non-SC/ST group (AOR = 1.36, *p<0*.*01*) were more likely to be overweight/obese as against SC/ST women. Similarly, odds of being overweight/obese were high among non-Hindus (AOR = 1.33, *p<0*.*01*) as compared to Hindus. Urban women had almost 50% more chances (AOR = 1.52, *p<0*.*01*) to be overweight or obese in comparison to their rural counterparts. Women from non-poor economic groups were 2.6 times more likely (AOR = 2.62, *p<0*.*01*) to be overweight/obese as against women from poor groups. Similarly, women who has caesarean had 58% higher chances to be overweight/obese (AOR = 1.58, *p<0*.*01*) than women who never had caesarean. Women who ever had their pregnancy terminated also had higher odds (AOR = 1.18, *p<0*.*01*) to be overweight or obese as opposed to their counterparts. Women who watch TV have also higher chances to be overweight/obese (AOR = 1.42, *p<0*.*01*) as compared to those who do not watch TV. Women consuming fast foods has only 4% higher chances (AOR = 1.04, *p<0*.*01*) of being overweight/obese as against those not consuming fast-foods.

**Table 2:** Results from bivariate and logistic regression analysis for overweight/obesity among women by background factors in India, 2015–16.

Background characteristics	Overweight/obesity (%)	AOR (95% CI)
**Age (in years)**		
15–29	14.7	Ref.
30–49	30.5	2.91***(2.86–2.97)
**Education**		
No or primary	19.2	Ref.
Secondary or higher	30.1	1.29***(1.27–1.31)
**Caste**		
SC/ST	27.7	Ref.
Non-SC/ST	18.0	1.36***(1.33–1.38)
**Religion**		
Hindu	23.7	Ref.
Non-Hindu	30.3	1.33***(1.31–1.35)
**Place of residence**		
Urban	37.8	1.52***(1.5–1.55)
Rural	18.3	Ref.
**Wealth**		
Poor	10.5	Ref.
Non-Poor	33.4	2.62***(2.56–2.67)
**Ever had caesarean**		
No	24.4	Ref.
Yes	31.1	1.58***(1.54–1.63)
**Ever had terminated pregnancy**		
No	24.1	Ref.
Yes	28.9	1.18***(1.16–1.21)
**Watching TV**		
No	12.0	Ref.
Yes	28.9	1.42***(1.38–1.45)
**Eats fast food**		
No	23.3	Ref.
Yes	26.2	1.04***(1.02–1.06)

Ref: Reference category; AOR: Adjusted odds ratio; CI: Confidence interval; TV: Television; SC/ST: Scheduled caste/scheduled tribes

The values of univariate and bivariate Moran’s I statistics are depicted in **Table 3**. Univariate Moran’s I statistics reflect the spatial auto-correlation of outcome and explanatory variables. The value of spatial-autocorrelation for overweight/obesity was 0.64 depicting higher dependence of the outcome variable on districts of India. Further, women who consume fast-food (0.77), who were non-Hindu (0.72) or non-poor (0.70) had the highest Moran’s I values among explanatory variables. There was negative spacial auto-correlation between overweight/obesity and consumption of fast-foods (-0.03). The spatial auto-correlation of overweight/obesity with non-Hindus was 0.10, and that with non-poor was 0.57. In addition, spatial auto-correlation of overweight/obesity with age above 30 was 0.33, with secondary or higher education was 0.45 and with women watching TV was 0.48.

**Table 3:** Univariate and Bivariate Moran’s I values for the outcome and explanatory variables for women in India, (N = 640).

Predictors	Univariate	Bivariate
		Overweight/obesity
Overweight/obesity	0.64 (0.001)	----
Age above 30 years	0.43 (0.001)	0.33 (0.001)
Secondary or higher education	0.65 (0.001)	0.45 (0.001)
Non-SC/ST	0.56 (0.001)	0.16 (0.001)
Non-Hindu	0.72 (0.001)	0.10 (0.001)
Urban	0.40 (0.001)	0.30 (0.001)
Non-Poor	0.70 (0.001)	0.57 (0.001)
Ever had caesarean	0.58 (0.001)	0.46 (0.001)
Ever had terminated pregnancy	0.47 (0.001)	0.08 (0.001)
Watching TV	0.67 (0.001)	0.48 (0.001)
Eats fast food	0.77 (0.001)	-0.03 (0.04)

**Table 4** depicts the estimates from spatial regression model for overweight/obesity and its determinants across 640 districts in India. The estimates from OLS re-affirmed that women’s age above 30 years (β = 0.217, *p<0*.*01*), non-poor (β = 0.231, *p<0*.*01*), ever having a caesarean (β = 1.041, *p<0*.*01*), ever terminating pregnancy (β = 0.171, *p<0*.*01*), and watching TV (β = -0.078, *p<0*.*01*) were significant spatial predictors of overweight/obesity among women in India. The values of adjusted R-square and AIC were found to be 0.78 and 3659.0 respectively.

**Table 4:** Spatial regression model for estimating the spatial association between overweight/obesity by background factors among women in India, (N = 640).

Predictors	Overweight/obesity		
	OLS	SLM	SEM
Age above 30 years	0.217 (0.000)	0.21 (0.000)	0.159 (0.000)
Secondary or higher education	0.025 (0.168)	0.021 (0.205)	0.006 (0.784)
Non-SC/ST	-0.004 (0.639)	-0.001 (0.874)	0.005 (0.610)
Non-Hindu			
Urban	0.026 (0.011)	0.043 (0.000)	0.056 (0.000)
Non-Poor	0.231 (0.000)	0.174 (0.000)	0.196 (0.000)
Ever had caesarean	1.041 (0.000)	0.856 (0.000)	0.65 (0.000)
Ever had terminated pregnancy	0.171 (0.000)	0.134 (0.000)	0.084 (0.033)
Watching TV	-0.078 (0.000)	-0.069 (0.000)	-0.033 (0.077)
Eats fast food	0.007 (0.423)	0.006 (0.505)	-0.013 (0.279)
**N**	640	640	640
**ρ**		0.28 (0.000)	
**Lamda**			0.732 (0.000)
**AIC**	3659.0	3560.0	3428.0
**Adjusted R**	0.78	0.82	0.87

AIC: Akaike information criterion; OLS: Ordinary least-square; SLM: Spatial lag model; SEM: Spatial error model; SC/ST: scheduled caste/scheduled tribe

As per the results from the SLM, the lag coefficient for overweight/obesity was found to be 0.28 (*p< 0*.*01*) implying that a change in the prevalence of overweight/obesity in a certain district may statistically lag the prevalence of overweight/obesity by 28% in the neighboring districts. Keeping all the variables constant, the findings from SLM further suggested that unit increase in women’s age was associated with a 0.21 increase in overweight/obesity among women (β = 0.21, p<0.01). Similarly, unit increase in urban place of residence was associated with a 0.043 increase in outcome variable (β = 0.043, *p<0*.*01*). Additionally, unit increase in non-poor quintile was associated with 0.174 increase in overweight/obesity among women (β = 0.174, *p<0*.*01*). Result also show that unit increase in caesarean delivery and pregnancy terminating was associated with a 0.856 and 0.134 increase respectively, in overweight/obesity among women. Moreover, unit increase in watching TV was associated with a decrease in overweight/obesity (β = -0.069, *p<0*.*01*). The value of adjusted R-square was 0.82 and AIC was found to be 3560.0.

Nevertheless, the theory of spatial regression models suggests that the model with the lowest AIC and highest R-square value should be considered as the best fit model. Consequently, in this case, the spatial error model (SEM) can be considered as the best fit model, as it had the lowest AIC (3428.0) and highest R-square (0.87) values among all three models. Further, the value of spatial autoregressive coefficient/error lag value (lambda) was 0.732 (*p<0*.*01*), which reflected that the spatial influence on overweight/obesity prevalence through omitted variables was absent in the SEM.

The results from SEM further depict that if in a district, women aged 30 years or more increased by 10%, the share of overweight/obese women increased by 1.6%. Similarly, in a district where women living in urban areas increased by 10%, the share of overweight/obese women increased by 0.6%. Likewise, if the share of women from non-poor quintiles increased by 10%, share of overweight/obese women increased by 2.0%. Ever having caesarean (β = 0.65, *p<0*.*01*) and ever having a terminated pregnancy (β = 0.084, *p<0*.*05*) were also significant factors having positive association with prevalence of overweight/obesity.

Additionally, secondary or higher education (β = 0.006, *p>0*.*05*); and non-SC/ST group (β = 0.005, *p>0*.*05*) had positive association with overweight/obese estimates, however the results were not significant. Furthermore, watching TV (β = -0.033, *p> 0*.*05*) and consuming fast-foods (β = -0.013, *p> 0*.*05*) had negative, though not significant association with overweight/obesity estimates. The results implied that districts with higher share of women in older age groups (30–49 years), living in urban area, from non-poor wealth quintiles, or those who ever had caesarean had more chances to have higher share of overweight/obese women.

**Fig 1:**
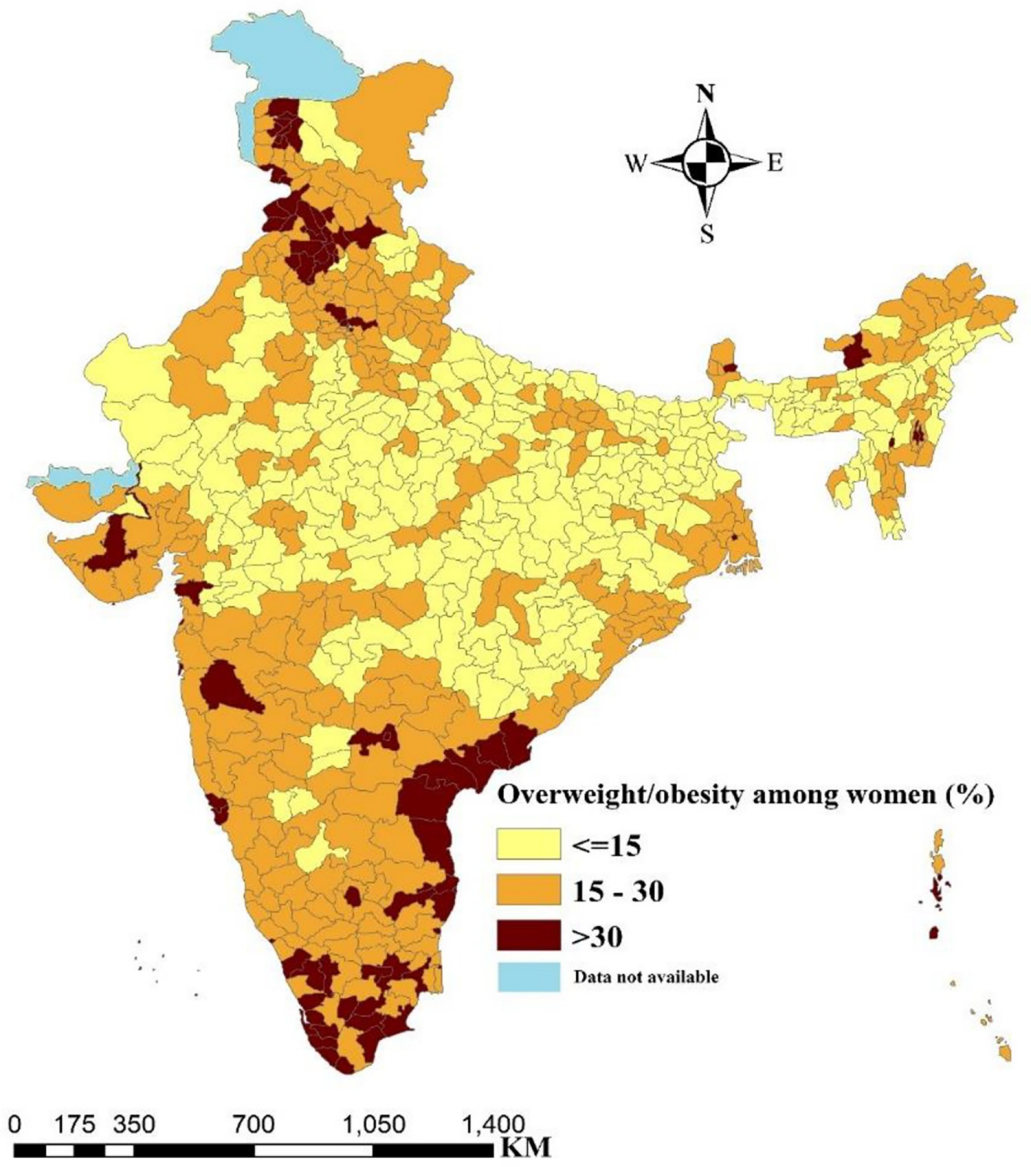
Percentage distribution of overweight/obesity among women in districts of India.

**Fig 1** depicts the spatial distribution of the overweight/obese women across all districts in India. The colour pattern illustrates the spatial differences in the distribution of overweight/obese women, where darker colour denotes areas with higher proportion of overweight/obese women and light colour indicates lower share of overweight/obese women. In many districts of Punjab, Kerala, Tamil Nadu and Andhra Pradesh, and a few districts in Gujarat, Maharashtra and Jammu & Kashmir, more than 30% of women aged 15–49 years are overweight or obese.

**Fig 2:**
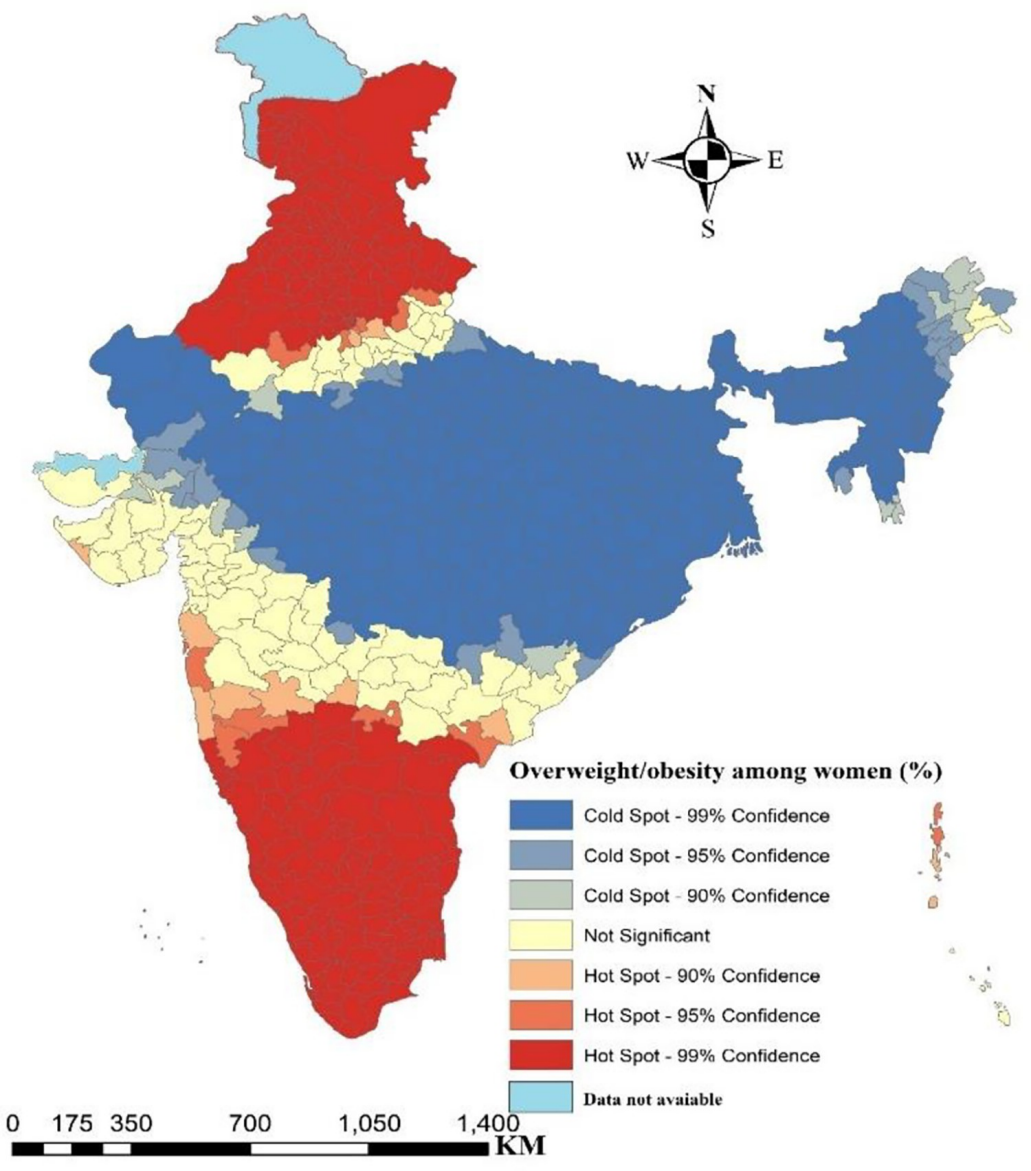
Hotspot map for overweight/obesity among women in districts of India.

**Fig 2** depicts the hotspot analysis of overweight/obese women across India. The map clearly reflects that the northern India, comprising of states viz. Punjab, Haryana, Himachal Pradesh, Delhi, Uttarakhand, parts of Rajasthan, Jammu and Kashmir and southern part of India including states such as Tamil Nadu, Kerala and parts of Andhra Pradesh and Karnataka have high concentration of overweight/obese women.

**Fig 3:**
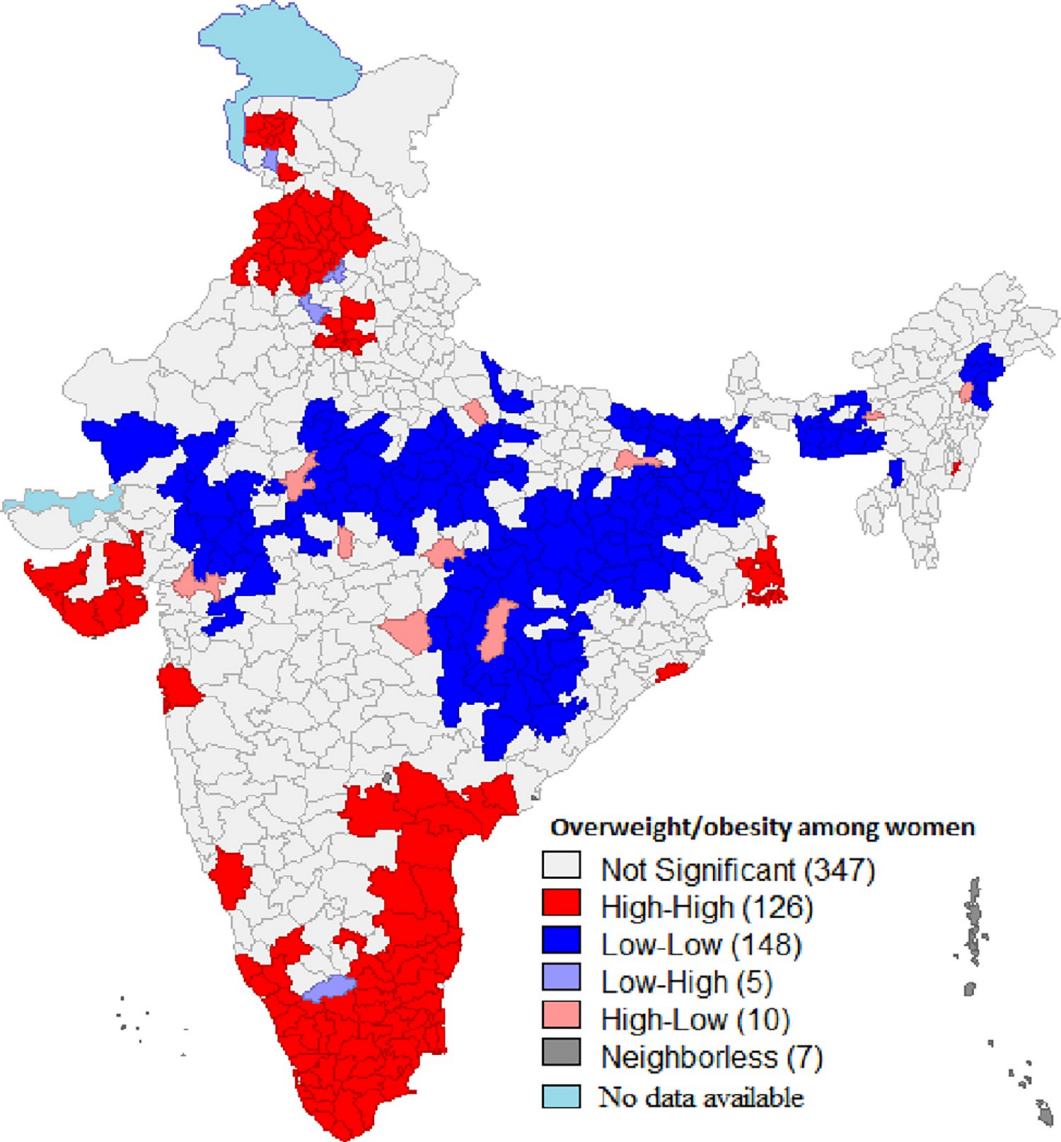
Univariate LISA map overweight/obesity among women in India, (N = 640).

**Fig 3** reflects univariate LISA (cluster and significance) map for the overweight/obese women in the reproductive age-group. A significant high-high clustering was found in 126 districts from states such as Kerala, Tamil Nadu, Punjab and parts of Himachal Pradesh, Haryana, Uttar Pradesh, Gujarat, Andhra Pradesh, Odisha and Karnataka. The figure also depicts 148 cold-spots (low-low clusters) located in states such as Madhya Pradesh, Bihar, Jharkhand, Chhattisgarh, and parts of Rajasthan.

**Fig 4:**
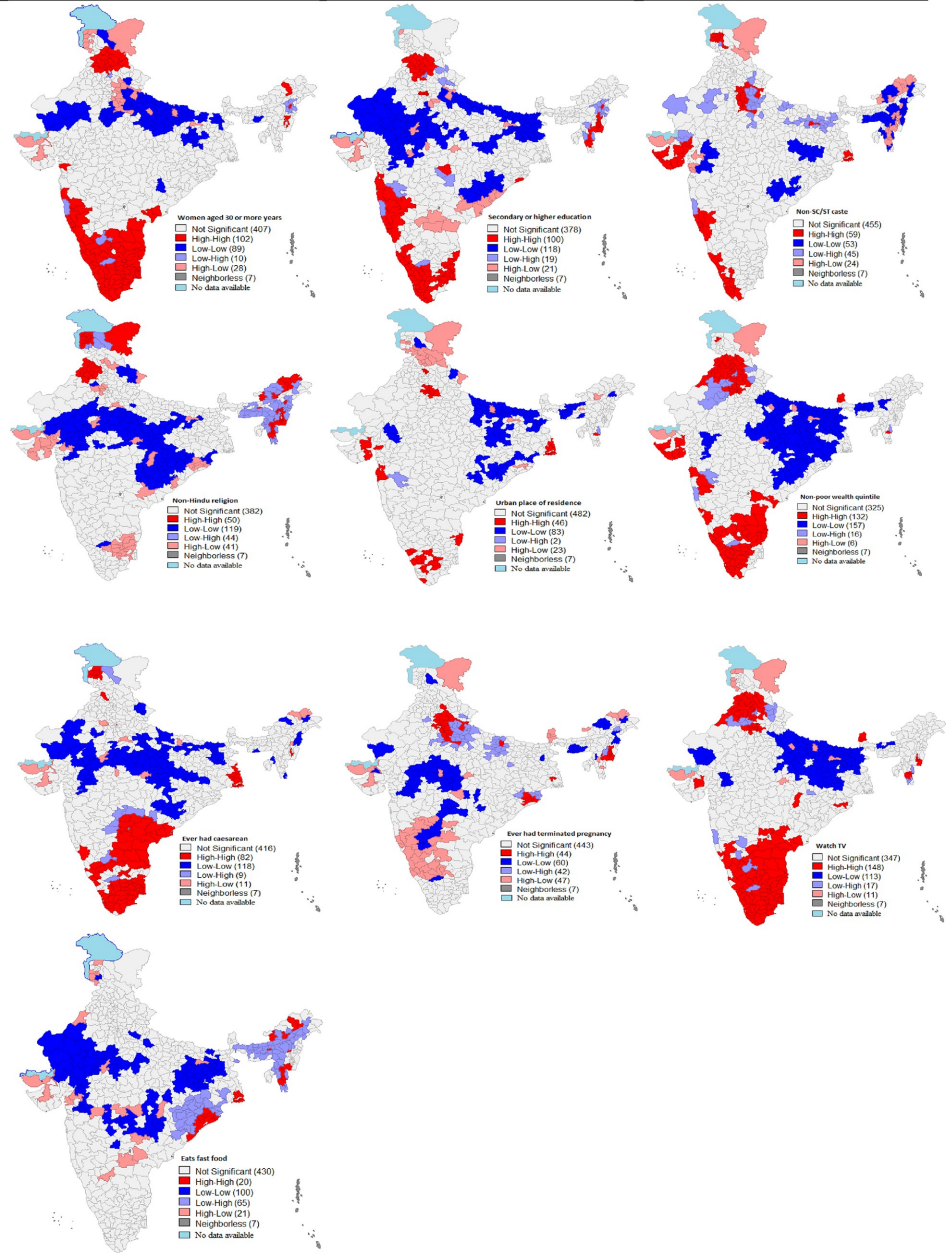
Bivariate LISA maps for overweight/obesity among women by background characteristics, India (N = 640).

**Fig 4** shows the bivariate LISA cluster maps for overweight/obese women in reproductive age-group by background characteristics of women. The figure shows high-high clusters of women aged 30 years and more who are overweight/obese in 102 districts, mostly from southern part of the country and some districts in Punjab, Himachal Pradesh, Maharashtra and Andhra Pradesh. Women who were overweight/obese and had secondary or higher education had higher concentration in 100 districts across India. There were 59 hotspots (high-high clusters) for women from non-SC/ST class who were either overweight or obese, with majority districts from Gujarat, Maharashtra, Kerala, and Delhi National Capital Region. About 46 out of 640 districts had the high clustering of overweight/obese women and urban place of residence. There were significantly high clustering of overweight/obese women and non-poor wealth quintiles in 132 districts, mainly from states of Punjab, Haryana, Gujarat, Maharashtra, Kerala, Tamil Nadu, Karnataka and Andhra Pradesh. There were high-high clustering of overweight/obese women and those who ever had caesarean in 82 districts, mostly from Kerala, Tamil Nadu, Andhra Pradesh and Karnataka. There were 44 districts with high-high clusters of overweight/obese women and those who ever had their pregnancy terminated, with majority of districts from Delhi, Haryana, western UP and Uttarakhand. There were 148 hotspots with high-high clustering of overweight/obese women and those who watched television. Most of these districts were from Punjab. Haryana, Kerala, Tamil Nadu, Karnataka and Andhra Pradesh.

## Discussion

The study highlights the regional aspect of the burden of overweight and obesity and controls for the socio-demographic variabilities, in Indian context. Approximately 25 percent of women in the reproductive age-group in India, that has doubled from the past NFHS survey rounds, are either overweight or obese and have crossed the global average [40]. Overweight and obesity might be a problem of the developed countries but the epidemic has started to take its toll on the developing countries too. In India, while double burden of malnutrition and hunger already exists, the burden of overweight and obesity is also rising [41]. Various studies attribute this rise to the transition from traditional diets to westernized diets in developing nations. Moreover, transition in diets coupled with overall adaptation of western culture that comes with sedentary lifestyle, consumption of fast food, stressful life and environmental pollution lead to obesity [13, 42, 43].

Older age has a significant association with overweight or obesity, i.e., aging increases the risk of increasing BMI. Women with higher number of children, or those who had caesarean delivery and had terminated pregnancy have higher risk of being overweight or obese. The pregnancy weight gain that often sustains a lifetime is also an attributable cause and is also a highly associated factor with caesarean delivery [21, 44–46]. Prevalence of overweight or obesity is evidently more among higher educated women, also corroborated by other studies [9, 47–49]. Furthermore, the socio-economic variables such as urban residence, higher wealth index and higher social status, also had significant associations with being overweight or obesity. Higher levels of education bring about better livelihood opportunities for women which promotes self-dependency and higher socio-economic status [50]. The findings show that rich people are more prone to be overweight or obese than poor ones. Higher purchasing ability allows them to get everything at doorstep without much movement and also follow unhealthy dietary patterns which is reflected from the study result as well, where the likelihood of becoming overweight or obese is high among those who eat more fast foods [51, 52]. All of these factors leads to weight gain and ultimately becoming overweight or obese. The socio-economic and demographic variabilities are attributable to the differences in the categories of body weights in various population subgroups. For instance, some studies suggest that people belonging to the Hindu religion in India are mostly vegetarians who consumes less calorie food as compared to people of other religions like Muslims or Christians, hence they have lower prevalence of overweight or obesity [53, 54].

The spatial patterns on the prevalence of overweight and obesity in India showed that the women belonging to the southern states are more overweight or obese in comparison to other states. Some of the possible reasons could be that the socio-economic status and female literacy rates in South Indian states are much higher [55, 56]. Additionally, women living below poverty line are much less in proportion in the Southern states in comparison to other states [57]. In India, southern states are much ahead in terms of women empowerment and they enjoy a much better life with good education and income which again brings in the factor of sedentarism and unhealthy lifestyle practices [50]. The framework by Haddad L. et al. [58] showed that factors affecting under and over-nutrition (in our case overweight/obesity), can be categorized into three levels, which includes- immediate (health and biological factors); underlying (social and environmental factors); and basic (economic and political). The interaction among these factors result in the nutrition level to be over or under. The increasing urbanization along with better income has led to more access to high calorie food intake, processed food and lack of physical activity, which causes obesity and related diseases [13, 18]. Cultural and societal factors significantly influence dietary habits and lifestyle choices in the southern region of India. A shift from traditional diets to more westernized dietary patterns, characterized by increased consumption of refined carbohydrates, unhealthy fats, and added sugars, has contributed to the rising burden of overweight and obesity [26]. Overweight and obesity among women in the southern region of India have severe health consequences. Studies have highlighted the increasing risk of chronic conditions associated with excess weight. For instance, a study conducted in Kerala found a positive association between obesity and the prevalence of hypertension and type 2 diabetes among women [59].

The study findings have significant public health implications by providing empirical evidences on scenario of overweight or obesity among women in India. In a country which comprises of more than one-twelfth of the global women population, with half of them being in reproductive age-group, the results of the study give sufficient representation of national scenario. Women are at elevated risks of developing complications due to high BMIs resulting in becoming overweight or obese, mostly due to the identified lifestyle factors. It is necessary to increase awareness and educate them on the importance of maintaining a healthy weight which will stem from practicing healthy lifestyle. Indian health programmes and policies that focus on the marginal and vulnerable populations of the societies are many a times pro-rural, often ignoring the issue of overweight and obesity, which is mostly a problem in the urban areas. There is a need to focus on the growing epidemic of overweight/obesity among urban women, which will further prevent the burden of chronic conditions like diabetes, cardiovascular diseases, hypertension and infertility in India. The health programmes can promote healthy diet and physical exercises by incorporating them into special clauses. However, we can consider the policy implications from the study such as implementing comprehensive health education programs to increase awareness about the risks and consequences of overweight and obesity, targeting women through community-based initiatives, schools, and healthcare facilities to promote knowledge about healthy lifestyles, ensuring early screening detection and intervention for overweight and obesity-related health conditions; incorporating weight-related screenings and counseling into routine healthcare check-ups for women.

The study has certain limitations such as, the data being cross-sectional, there is a lack of evidence about temporal relationship due to which some observed factors might not be causally associated. The survey has limited information on the lifestyle habits that includes physical activity and sleep patterns and walkability. However, the study captures the socio-demographic, economic, lifestyle and pregnancy related factors that determine overweight and obesity, a more detailed study on subjects based on these correlates can help in gaining better understanding.

## Conclusion

The present study depicts the spatial patterns and clusters of overweight or obesity among women in India. Determinants like older age, higher education, urban residence, higher economic status, are the important factors contributing to the prevalence of overweight or obesity among women in the reproductive age groups. The study results also reveal a huge proportion of women belonging to the BMI categories of non-normal, which is a concern and can increase the risks of developing non-communicable diseases. Hence, the study concludes and recommends an urgent need of interventions catering to urban women belonging to higher socio-economic status, which can reduce the risks of health consequences due to overweight and obesity. Development of nutrition specific as well as sensitive interventions can be done for mobilization of local resources that addresses the multiple issues under which woman is overweight or obese. Also, an urgent need for focus on the women from non-poor wealth quintile is suggested so that the burden of overweight and obesity can be reduced among them.

Reiterating the importance of policy interventions to address the health outcomes and long-term impact of overweight and obesity among women in India, there is a need for multi-sectoral collaboration and sustained efforts to promote healthy lifestyles and reduce the burden of overweight and obesity in this population. These policy implications provide a starting point for addressing overweight and obesity among women in India. It is crucial to adapt and tailor interventions to the local context, considering socio-cultural factors, regional disparities, and the diverse needs of women across different socioeconomic groups and geographic regions.
